# Higher Expression of DNA (de)methylation-Related Genes Reduces Adipogenicity in Dental Pulp Stem Cells

**DOI:** 10.3389/fcell.2022.791667

**Published:** 2022-02-24

**Authors:** Adaylu A. Argaez-Sosa, Beatriz A. Rodas-Junco, Leydi M. Carrillo-Cocom, Rafael A. Rojas-Herrera, Abel Coral-Sosa, Fernando J. Aguilar-Ayala, David Aguilar-Pérez, Geovanny I. Nic-Can

**Affiliations:** ^1^ Facultad de Ingeniería Química, Campus de Ciencias Exactas e Ingeniería, Universidad Autónoma de Yucatán, Mérida, Mexico; ^2^ Laboratorio Translacional de Células Troncales, Facultad de Odontología, Universidad Autónoma de Yucatán, Mérida, Mexico; ^3^ CONACYT-Facultad de Ingeniería Química, Campus de Ciencias Exactas e Ingeniería, Universidad Autónoma de Yucatán, Mérida, Mexico

**Keywords:** adipogenesis, cell differentiation, dental pulp stem cells, DNA methylation, periodontal ligament stem cells

## Abstract

Obesity is a significant health concern that has reached alarming proportions worldwide. The overconsumption of high-energy foods may cause metabolic dysfunction and promote the generation of new adipocytes by contributing to several obesity-related diseases. Such concerns demand a deeper understanding of the origin of adipocytes if we want to develop new therapeutic approaches. Recent findings indicate that adipocyte development is facilitated by tight epigenetic reprogramming, which is required to activate the gene program to change the fate of mesenchymal stem cells (MSCs) into mature adipocytes. Like adipose tissue, different tissues are also potential sources of adipocyte-generating MSCs, so it is interesting to explore whether the epigenetic mechanisms of adipogenic differentiation vary from one depot to another. To investigate how DNA methylation (an epigenetic mark that plays an essential role in controlling transcription and cellular differentiation) contributes to adipogenic potential, dental pulp stem cells (DPSCs) and periodontal ligament stem cells (PLSCs) were analyzed during adipogenic differentiation *in vitro*. Here, we show that the capacity to differentiate from DPSCs or PLSCs to adipocytes may be associated with the expression pattern of DNA methylation-related genes acquired during the induction of the adipogenic program. Our study provides insights into the details of DNA methylation during the adipogenic determination of dental stem cells, which can be a starting point to identify the factors that affect the differentiation of these cells and provide new strategies to regulate differentiation and adipocyte expansion.

## Introduction

During periods of food surplus, animals store excess energy in the form of triglycerides within a large unilocular droplet, which is confined to specialized cells called white adipocytes. Unfortunately, an imbalance between caloric intake and energy expenditure promotes an increases the size of the existing adipocytes (hypertrophy) or promotes the formation of new adipocytes (hyperplasia), which can lead to the development of obesity and impair metabolic health (Shook et al., 2016). White adipose tissue is organized into two distinct depots: visceral and subcutaneous depots. Other adipocyte niches include the dermis, bone marrow, pericardium, cranial area and skeletal muscle (Sanchez-Gurmaches et al., 2016; Hepler and Gupta 2017), which have attracted increasing interest because every niche can contain unique adipocyte precursor cells with different markers or adipogenic capabilities (Ghaben and Scherer 2019). The generation of adipocytes is triggered by signaling factors that induce the transition of mesenchymal stem cells (MSCs) into preadipocytes that eventually mature into adipocytes, which can synthesize, store and transport lipids. At the molecular level, adipogenic stimuli promote the activation of two transcriptional waves involving a large number of transcription factors (TFs). The first wave leads to the expression of the CCAAT/enhancer-binding protein *C/EBPβ* and *-δ,* Krüppel-like factors (*KLF4* and *KLF5*), cAMP-response element-binding protein (CREB), the glucocorticoid receptor and activator of transcription 5A, which in turn activate TFs of the second wave, including peroxisome proliferator-activated receptor-γ (PPARγ) and *C/EBPα,* which are considered the most prominent key factors that lead to the terminal adipocyte phenotype (Siersbaek et al., 2011; Lefterova et al., 2014). These important findings have been mainly obtained from the mouse cell lines C3HT10T1/2 and 3T3-L1 for studying commitment or adipocyte terminal differentiation, respectively (Tang and Lane 2012). More recently, MSCs derived from human bone marrow (BMMSCs), umbilical cord (UCMSCs) and adipose tissue (ATMSCs) or induced pluripotent stem cells (iPSCs) under appropriate stimuli can generate adipocytes (Taura et al., 2009; Liu et al., 2017). Unfortunately, mice and humans differ in some aspects of adipose biology (Hepler and Gupta 2017), whereas MSCs from different depots may vary in their ability to differentiate toward exclusive lineage cells due to their transcriptomic and epigenetic profiles. In this regard, DNA methylation (the presence of a methyl group at C5 of 2′-deoxcycytosine, 5mC), which is dispersed through chromatin and inherited during successive cell cycles, provides a means to regulate the gene expression that defines a specific cell lineage (Luo et al., 2018). Methylation is written by DNA methyltransferases (DNMT1, -3A and -3B) but can be reversed by ten-eleven translocation proteins (TET1-3). Thus, DNA methylation-related enzymes play a key role in gene regulation and cellular differentiation. However, the role of dynamic DNA methylation in adipogenesis appears to vary among species (Ruiz-Ojeda et al., 2016; Hepler and Gupta 2017). Moreover, the epigenetic memory partially imposed by tissue-specific DNA methylation patterns determines the differentiation potential of human MSCs. Although mouse cell models have provided new clues about adipocyte origins, such discoveries await validation in new experimental systems.

In this regard, human dental tissue-derived MSCs (hDT-MSCs) have emerged as a promising source for cellular therapy and regenerative treatment due to their multilineage potential (Liu et al., 2015). The ability of hDT-MSCs to differentiate into different cell types, including pancreatic cells, neural cells or adipocytes, is due to the retention of embryogenic features conferred through their origin from neural crest cells (NCCs) (La Noce et al., 2014; Dunaway et al., 2017). Among hDT-MSCs, dental pulp and periodontal ligament stem cells (DPSCs and PLSCs, respectively) can be easily obtained from a single tooth extraction without ethical concerns, which facilitates their use to study cell fate and differentiation. Although DPSCs and PLSCs exhibit strong differentiation potential, studies have mainly focused on their osteogenic capacity in the context of bone defect restoration (Paino et al., 2017; Yang et al., 2017; Zhou et al., 2020), and little or no attention has been given to their adipogenic differentiation. Here, we propose that both DPSCs and PLSCs offer an excellent model to study adipogenic commitment, since both stem cell populations, similar to adipocytes, originate from NCCs. In this study, we discuss the particularities regarding to DNA methylation-related genes and their influence on lineage decisions when DPSCs and PLSCs are induced to undergo adipogenic differentiation.

## Results

### Stemness- and Adipogenic-Related Genes Differ Between DPSCs and PLSCs Under Adipogenic Culture Conditions

**FIGURE 1:**
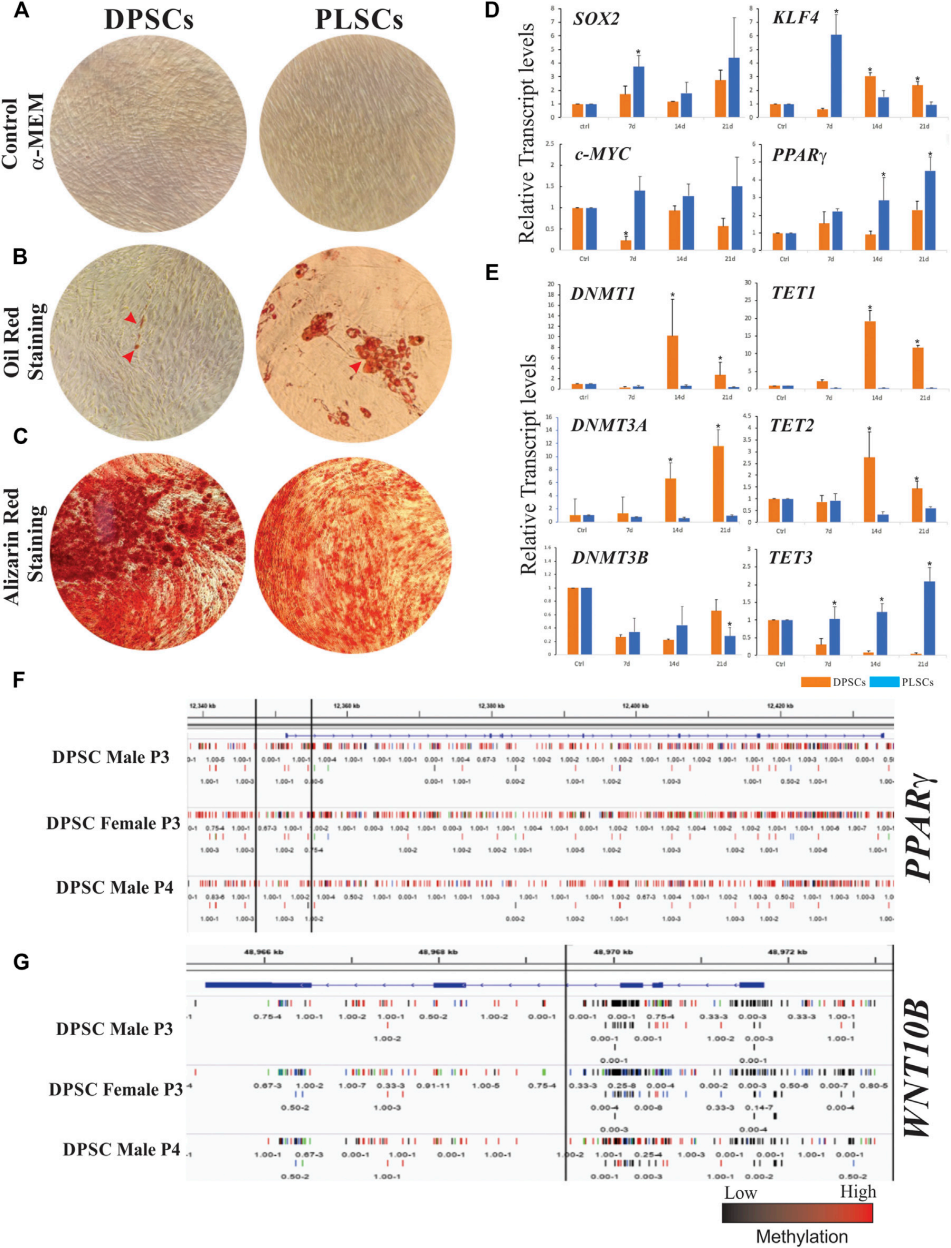
Cellular differentiation and gene expression patterns of dental stem cells. Morphology of human dental pulp stem cells (DPSCs) and periodontal ligament stem cells (PLSCs) cultured in α-MEM medium as a control **(A)**. Adipogenic and osteogenic capacity of DPSCs and PLSCs determined by Oil Red and Alizarin Red staining, which show lipid droplets in the adipogenically induced culture for 21 days and calcified mineralized matrix after 14 days of induction, respectively **(B**–**C)**. Gene expression profiles of stemness- and adipogenesis-related genes **(D)** and DNA methylation-related genes **(E)** in cultured DPSCs and PLSCs (orange and blue bars, respectively) for 0, 7, 14 and 21 days under adipogenic conditions. Representative genomic regions of PPAR*γ* **(F)** and WNT10B **(G)** in DPSCs, which show methylation differences over the gene structure. Different tracks represent the methylation of three biological replicates from an analysis of previously published raw data (Dunaway et al., 2017). The error bars represent the standard deviation of the mean from three independent experiments. The asterisks (*) represent the significance of differences with respect to the control (*p* < 0.05).

In a previous study, we reported that DPSCs and PLSCs exhibited similar morphology and expression patterns to MSCs; however, DPSCs exhibit a poor adipogenic response compared to other hDT-MSCs (Mercado-Rubio et al., 2021) such as PLSCs (Figure 1B). The commitment of MSCs to adipocyte differentiation involves several transcription factors that regulate the expansion of pre-adipocytes and the generation of lipid droplets. Hence, the expression levels of stemness- (*SOX2* and *c-MYC*) and adipogenesis-related genes (*KLF4* and *PPARγ*) in DPSCs and PLSCs were profiled by RT–qPCR (Figure 1D). The expression of *SOX2* and *c-MYC* was mainly upregulated in PDLSCs compared to DPSCs, by > 2- and 6-fold during the first week of induction. Furthermore, DPSCs exhibited late expression of *KLF4* under adipogenic induction, whereas the expression in PLSCs was > 8-fold compared to DPSCs 7 days after induction (dai) with a gradual decrease toward 21 dai. Interestingly, gradually increased expression of the key adipogenic factor *PPARγ* was observed in PLSCs but not in DPSCs during adipogenic induction. These findings are consistent with the strong capacity of PLSCs compared to DPSCs to synthesize lipids, as demonstrated by multilocular droplets staining with Oil Red (Figure 1B).

### The Difference in Adipogenic Capacity Between DPSCs and PLSCs Is Linked to the Differential Expression of DNA Methylation-Related Genes

Because changes in DNA methylation are dynamic and may contribute to lineage differentiation (Rinaldi and Benitah 2015), we reasoned that the expression of DNA methylation-related genes could help explain the differential adipogenic potential of DPSCs and PLSCs. Our results show that the expression of *DNMT1* in DPSCs was >16- and 8-fold higher than that in PLSCs, whereas the expression of *DNMT3A* exhibited a gradual increase in DPSCs but not in PLSCs during adipogenic induction. In contrast, downregulation of the *DNMT3B* expression was observed in both hDT-MSCs analyzed (Figure 1E). Considering this behavior and by using previous DPSC methylome data (Supplementary material) (Dunaway et al., 2017), we found that the low expression of *PPAR*γ in our study (Figure 1D) might be related to the hypermethylation of DNA, which extended from the promoter to all coding regions of this gene (Figure 1F) probably mediated by DNMT1 and -3A, as indicated by our results (Figure 1E). In contrast, hypomethylation extending from the promoter to the first and second exons of the *WNT10B* gene (Figure 1G) could stimulate its expression and favor osteogenesis over adipogenesis. Since TET family proteins are involved in the removal of 5mC from DNA, the mRNA levels of *TET1-3* were also investigated. The expression of *TET1* and *TET2* significantly increased in DPSCs compared with PLSCs at 14 and 21 dai (e.g., between 48- and 28-fold and between 7.8- and 2.28-fold, respectively). In contrast, *TET3* was upregulated between 2.5- and 50-fold in PLSCs compared to DPSCs under adipogenic conditions (Figure 1E).

## Discussion

Isolated DPSCs and PLSCs have recently emerged as a valuable source for endodontic regeneration, therapeutics and regenerative treatments (Zhou et al., 2020; Li et al., 2021) due to their multilineage capacity. However, the microenvironment of the tissue source affects the fate and degree of lineage commitment of MSCs. For example, although they are immunophenotypically and morphologically similar to several MSCs, including PLSCs and ATSCs (Xu et al., 2017), DPSCs exhibit limited or no capacity to differentiate into adipocytes according to recent reports (Kolar et al., 2017; Monterubbianesi et al., 2019; Shen et al., 2019; Fracaro et al., 2020). In support of these findings, the expression of TF-encoding genes required for the early adipogenic response, including *c-Myc, KLF4* and *PPARγ* (Deisenroth et al., 2014; Lefterova et al., 2014; Cervantes-Camacho et al., 2015), was downregulated in DPSCs but not in PLSCs, resulting in the formation of small lipid droplets in DPSCs after adipogenic induction (Figures 1B,D). In contrast and consistent with their mineralization capacity and ability to form bone and dentine tissues (Tatullo et al., 2015; Okajcekova et al., 2020), DPSCs exhibited notable capacity for osteogenesis (Figure 1C). This result suggests that the adipogenic capacity of MSCs depends on their cellular sources and *in vivo* functions. This interpretation is consistent with a recent study that indicated that *PPARγ* expression levels determine the adipogenic potential of ATMSCs, BMSCs and UCMSCs (Liu et al., 2017). Otherwise, although the loss of function of *KLF4* appears to have little effect on adipogenesis (Park and Ge 2017), KD of *c-MYC* inhibits adipogenesis by decreasing the *PPARγ, C/EBPβ, ADIPOQ* or *LPL* expression, which suggests that its functional role is necessary to modulate adipocyte maturation (Deisenroth et al., 2014). This finding exposes a similar scenario in DPSCs, where the decreased *c-MYC* expression sequentially affects the *PPARγ* expression (Figure 1D), *ADIPOQ* and *LPL* (Mercado-Rubio et al., 2021). Further work is necessary to address the mechanism by which this occurs. In addition, SOX2 inhibition alters the differentiation ability of BMSCs and UCMSCs by inhibiting adipogenic differentiation but enhancing the osteogenic differentiation ability (Park et al., 2012; Seo et al., 2013). These findings are consistent with our results, where *SOX2* is downregulated in DPSCs and exhibits strong osteogenic ability but poor adipogenic ability with respect to PLSCs. This result suggests that in addition to its role in stemness maintenance, SOX2 may significantly affect PPARγ activity.

Meanwhile, the low observed expression levels of adipogenesis-related genes, particularly *PPARγ,* in DPSCs (Monterubbianesi et al., 2019; Mercado-Rubio et al., 2021) cause a failure to activate the transcription of genes that initiate the terminal differentiation or maintenance of mature adipocytes. In support of this notion, a recent study disclosed that when cultured under adipogenesis, DPSCs displayed overrepresented genes that suppressed adipogenesis, such as *WNT10B* (Fracaro et al., 2020), whose protein product can prevent the expression of *PPARγ* and *C/EBP* family expression to stimulate osteogenic differentiation (Chen et al., 2016; de Winter and Nusse 2021). These results suggest that regardless of the MSC model, adipogenic differentiation requires more drastic transcriptional changes than osteogenesis (Rauch et al., 2019), which may be mediated by epigenetic mechanisms such as DNA methylation. The reason is that the patterns of 5-mC, which has been established during development, may be difficult to remove before generating a specific lineage different from the cell type of origin.

Unfortunately, although DNA methylation is often related to gene silencing, little is known about the expression of related genes in this process during the adipogenesis of DT-MSCs. Consistent with the adipogenic potential observed in ATMSCs and BMSCs, which depends on the DNA methylation at the CpG site of the *PPARγ* promoter (Xu et al., 2017), our results show that the upregulation of *DNMT1* and *DNMT3A,* but not *DNMT3B*, in DPSCs compared to PLSCs during the adipogenic induction (Figure 1E) can probably determine the differentiation capacity of DT-MSCs. In addition, the increased levels of DNA methylation-related genes in DPSCs correlate with the low expression of *SOX2*, *c-MYC*, *KLF4* and *PPARγ* (Figure 1D). These genes have been shown to be modulated by DNA methylation (Noer et al., 2006; Alonso et al., 2011; Yang and Zheng 2014) and may explain the limited adipogenic response in DPSCs (Figure 1B). In support of this idea, the DNMT1 silencing or inhibition of methylation upon treatment with 5-aza-2′-deoxycytidine (5-aza-dC) accelerates the adipogenic response in murine cells by increasing the expression of *BMP4* and decreasing the DNA methylation on the promoter of *PPARγ*, respectively (Bowers et al., 2006; Londono Gentile et al., 2013). Therefore, lower expression of *PPARγ* in DPSCs may reflect the hypermethylation of DNA observed at the locus of this gene (Figure 1F), which could be mediated by DNMT1 and -3A, as our results suggest (Figure 1E). However, there are still many controversies about the effect of DNA methylation on adipogenesis, and it is difficult to reach concrete conclusions.

On the other hand, the differentiation potential of DPSCs varies with the activity of DNMTs, which suggests that this epigenetic mark plays a critical role in the multilineage capacity of DPSCs (Zhou et al., 2020). For example, although DPSCs and PLSCs share similar DNA methylation patterns, slight differences in 5-mC in skeletal-related genes (e.g., SMAD and CD109) confer variations in osteogenesis capacity (Ai et al., 2018). In this regard, DPSCs exhibit higher mineralization capacity than other MSCs, including BMSCs, ATSCs, and different DT-MSCs (Xu et al., 2013; Padial-Molina et al., 2019; Shen et al., 2019; Mucientes et al., 2021). Thus, not surprisingly, the hypomethylation of the *WNT10B* locus (Figure 1G) facilitates its expression in DPSCs (Fracaro et al., 2020) to favor osteogenesis over adipogenesis, which suggests that DNA methylation is a mechanism underlying adipocyte and bone cell development. Together, these findings allow us to hypothesize that both the DNA methylation and gene expression established in DPSCs tend to be preserved, which suggests that the higher DNMT expression in DPSCs but not in PLSCs may be an important mechanism of cell identity that maintains the integrity of the epigenetic memory of DPSCs even under adipogenic conditions. Further investigations are needed regarding whether and how each DNMT blocks the commitment or adipogenic differentiation of DPSCs.

DNA demethylation family members also play an important role during the adipogenic differentiation of MSCs. However, their functionality may depend on the organism. TET1 and TET2 appear to have a positive effect in mice (Wiehle et al., 2016; Yoo et al., 2017; Qian et al., 2021); however, in human BMSCs, TET1 acts as a repressor of adipogenesis and osteogenesis (Cakouros et al., 2019). Consistent with this finding, the higher expression levels of *TET1* and *TET2* observed during adipogenic induction (Figure 1E) suggest another mechanism that prevents adipogenesis from protecting the genome integrity of DPSCs to maintain their mineralization capacity. This is supported by other studies showing that TET1 maintains repressed transcription of *PPARγ* and *RUNX2*, which are two essential master regulators of adipogenesis and osteogenesis commitment, but exhibits a swift response to change the fate of MSCs into osteoblasts by recruiting TET2 to active osteogenesis-related genes (Cakouros et al., 2019). Furthermore, TET1 enhances the odontogenic differentiation by modulating the *FAM20C* demethylation (Li et al., 2018), but its silencing by KD impairs the odontogenic differentiation in DPSCs (Li et al., 2014; Rao et al., 2016) and increases the adipogenic capacity of BMSCs (Cakouros et al., 2019), which reinforces its crucial role in mineralization events. Similar studies on TET2 KD have revealed that this demethylase has a dominant role in the osteogenesis of human ATSCs and BMSCs (Cakouros et al., 2019; Feng et al., 2020; Li et al., 2020), whereas the overexpression of this gene blocks adipogenic differentiation, which confirms that TET2 acts as an anti-adipogenic demethylase (Hou et al., 2020). Our results may help to indirectly confirm that both TET1 and TET2 act as repressors of adipogenesis, since the reduced expression of such demethylases correlates with the commitment of PLSCs to an adipogenic phenotype.

Finally, it is unknown whether TET3 has a role in adipogenesis. Its downregulation by KD has no significant effect on the adipogenic differentiation of BMSCs (Cakouros et al., 2019) or 3T3-L1 preadipocytes (Hou et al., 2020). Interestingly, in this study, we found that *TET3* was gradually expressed in PLSCs but not in DPSCs during adipogenic induction. This result suggests that TET3 may exhibit a tissue-specific function that contributes to modulating the cell fate of PLSCs by promoting adipocyte differentiation. Nonetheless, whether this demethylase can activate adipogenesis-related genes in PLSCs requires further investigation.

## Conclusions and Future Directions

**FIGURE 2:**
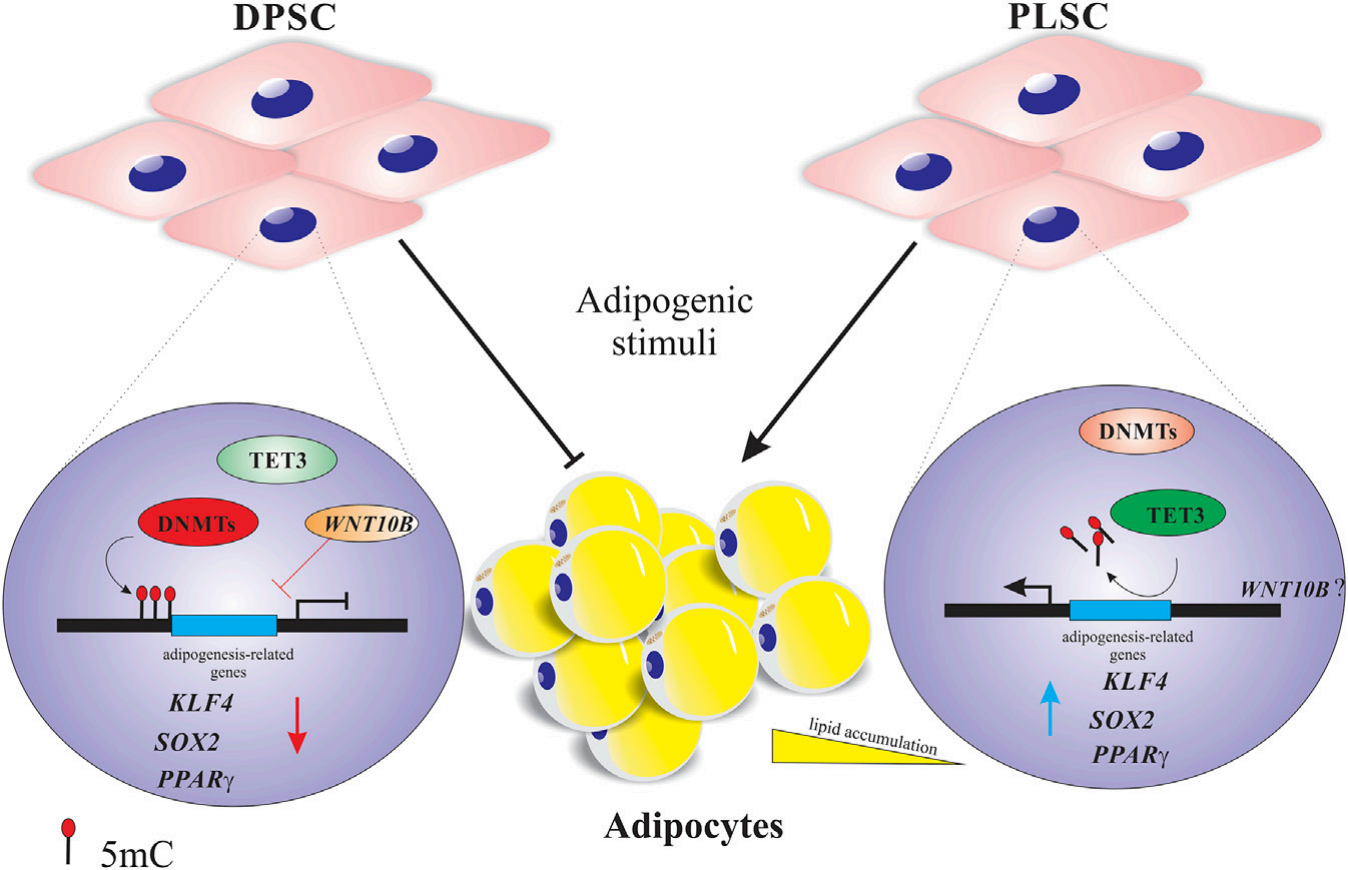
Proposed model of the dynamic DNA methylation of dental stem cells undergoing adipogenic differentiation. Higher expression of DNA methyltransferases (*DNMTs*) and demethylases (*TET1-2*) probably affects the down- and upregulation of adipogenesis-related genes (e.g., *PPARγ* or *KLF4*) and osteogenesis-related genes (e.g., WNT10B), respectively, to maintain the epigenetic memory of dental pulp stem cells (DPSCs) by repressing the adipogenic differentiation. Regarding periodontal ligament stem cells (PLSCs), the decreased expression of DNMTs and moderate upregulation of TET3 can influence the major expression of adipogenesis-related genes (e.g., *PPARγ* or *c-MYC)* and consequently their transition into mature adipocytes. The red and blue arrows indicate gene down- and upregulation.

Although dental tissue-derived mesenchymal stem cells hold great promise for tissue engineering and regenerative medicine, additional work is required to understand their true differentiation potential before selecting a suitable cell type for therapy. In this regard, we highlighted that the high expression levels of *DNMT1*, *-3A*, *TET1*, and *TET2* could prevent the transition of DPSCs, but not PLSCs, into adipocytes (Figure 2), which correlates with the downregulation of adipogenesis-related genes (*KLF4*, *c-MYC* and *PPARγ*)*.* This result suggests that these writers (DNMTs) and erasers (TETs) guarantee the integrity of the epigenetic memory of DPSCs by restricting fate changes to maintain their cell identity even under adipogenic conditions. However, we must not discard the possibility that the epigenetic regulation probably depends on the stem cell origin, culture conditions and method of adipogenic induction, among other factors.

Additional investigations are required to determine the particularities of 5mC and 5hmC in the variability of adipogenic response between DPSCs and PLSCs. In addition, it is of particular interest to reveal the molecular mechanisms by which DNMT1, -3A, TET1, and TET2 restrict adipocyte commitment in DPSCs and whether the overexpression of such genes impairs adipogenesis in responsive dental stem cells. In addition, recent data have demonstrated that TET1 interacts with TET2 or HDAC1 to facilitate osteogenesis or block the brown fat adipogenic program by preventing its DNA demethylase activity (Damal Villivalam et al., 2020). Thus, it will be interesting to determine whether the functional loss of *TET1* disrupts osteogenic commitment and improves the adipogenic response in DPSCs. Finally, although TET3 appears to have no significant role in the reported adipogenic systems, our results indicate a probable tissue-specific activity of this demethylase in PLSC adipogenesis. Additional studies are required to determine whether TET3 regulates adipogenesis-related genes. Overall, we believe that the functional validation of individual DNA methylation factors in DPSCs can offer lessons on how to avoid the induction of adipogenic precursor cells, which may result in therapies to help prevent obesity.

## Data Availability

The original contributions presented in the study are included in the article/Supplementary Material, further inquiries can be directed to the corresponding author.
