# Supplementary material for: Higher Expression of DNA (de)methylation-Related Genes Reduces Adipogenicity in Dental Pulp Stem Cells
Source: Front Cell Dev Biol. 2022 Feb 24;10:791667. doi: 10.3389/fcell.2022.791667 (PMC8907981; doi:10.3389/fcell.2022.791667)
Supplement: Supplementary file 1 [file DataSheet2.PDF]

Supplementary Table 1. *Primers sequences used for RT-qPCR*

Amplicon name	Sequence (F-5´-3´)	Sequence (R-5´-3´)	Annealing temperature	Amplicon size	References
18S ARNr	GGACAGGATTGACAGATTGAT	AGTCTCGTTCGTTATCGGAAT	60	111	This study
CD73	CAGCATTCCTGAAGATCCAAG	GATTGAGAGGAGCCATCCAG	56	120	(Bogdanova et al. 2014)
CD90	GTCCTCTACTTATCCGCCTTC	GACCAGTTTGTCTCTGAGCAC	56	123	(Bogdanova et al. 2014)
CD105	CTCAAGACCAGGAAGTCCATA	GATGAGGAAGGCACCAAAG	56	131	(Bogdanova et al. 2014)
DNMT1	GCAGCCATTAAGGAAGAC	TGGTTATTAGCGAAGAACATC	56	119	(Hara et al. 2013)
DNMT3a	TTACCTTACCATCGACCTCACA	CTGTCTCCATCTCCACTGTCT	56	150	(Hara et al. 2013)
DNMT3b	CCATCAGGCATTCTACCA	CGTTCTCCTTGTCTTCTCT	56	132	(Hara et al. 2013)
TET1	CGGTTTCACTTTTTACTTCAGGT	CAAGCGGAAGAATAACTCAACAAC	58	107	(Thienpont et al. 2016)
TET2	ATTCTCGATTGTCTTCTCTAGTGAG	CATGTTTGGACTTCTGTGCTC	58	99	(Thienpont et al. 2016)
TET3	GTGTAGATGACCTTCTCGATCC	TGCGTCGAACAAATAGTGGA	58	146	(Thienpont et al. 2016)
KFL4	TACCAAGAGCTCATGCCACC	CGCGTAATCACAAGTGTGGG	60	111	(Irie et al. 2015)
c-MYC	GGACCCGCTTCTCTGAAAGG	TAACGTTGAGGGGCATCGTC	60	104	(Irie et al. 2015)

SOX2	ACACCAA TCCCA TCCACACT	CCTCCCCAGGTTTTCTCTGT	60	117	(Irie et al. 2015)
PPARγ2	CTCCTATTGACCCAGAAAGC	TCAAAGGAGTGGGAGTGGTC	58	160	(Karaoz et al. 2011)

Supplementary References

Bogdanova A, Berzins U, Nikulshin S, Skrastina D, Ezerta A, Legzdina D, Kozlovska T. (2014). Characterization of human adipose-derived stem cells cultured in autologous serum after subsequent passaging and long term cryopreservation. *J Stem Cells* **9**: 135-148. doi:jsc.2014.9.3.135

Hara ES, Ono M, Eguchi T, Kubota S, Pham HT, Sonoyama W, Tajima S, Takigawa M, Calderwood SK, Kuboki T. (2013). miRNA-720 controls stem cell phenotype, proliferation and differentiation of human dental pulp cells. *PLoS One* **8**: e83545. doi:10.1371/journal.pone.0083545

Irie N, Weinberger L, Tang WW, Kobayashi T, Viukov S, Manor YS, Dietmann S, Hanna JH, Surani MA. (2015). SOX17 is a critical specifier of human primordial germ cell fate. *Cell* **160**: 253-268. doi:10.1016/j.cell.2014.12.013

Karaoz E, Demircan PC, Saglam O, Aksoy A, Kaymaz F, Duruksu G. (2011). Human dental pulp stem cells demonstrate better neural and epithelial stem cell properties than bone marrow-derived mesenchymal stem cells. *Histochem Cell Biol* **136**: 455-473. doi:10.1007/s00418-011-0858-3

Thienpont B, Steinbacher J, Zhao H, D'Anna F, Kuchnio A, Ploumakis A, Ghesquiere B, Van Dyck L, Boeckx B, Schoonjans L et al. (2016). Tumour hypoxia causes DNA hypermethylation by reducing TET activity. *Nature* **537**: 63-68. doi:10.1038/nature19081
