# Supplementary material for: Higher Expression of DNA (de)methylation-Related Genes Reduces Adipogenicity in Dental Pulp Stem Cells
Source: Front Cell Dev Biol. 2022 Feb 24;10:791667. doi: 10.3389/fcell.2022.791667 (PMC8907981; doi:10.3389/fcell.2022.791667)
Supplement: Supplementary file 2 [file DataSheet1.PDF]

1. **Supplemental experimental procedures:** Materials and Methods

1.1 **Isolation and cell culture**

Briefly, dental pulp and periodontal ligament tissues were obtained after informed consent from the patients from supernumerary teeth and third molars (n=2), respectively. Both oral tissues were cultured through the outgrowth method as described previously here (Mercado-Rubio et al. 2021). The cells were maintained in 10% fetal bovine serum (FBS, Gibco, USA) and 1% antibiotic (penicillin/streptomycin, Gibco, USA) and incubated at 37 °C with 5% CO_2. The medium culture was replaced twice a week. At approximately 80% confluence, the cells were collected with trypsin-EDTA 0.25% (Gibco, USA) and subcultured in a T-25 flask. Cells at passages 2-4 were used for the experiments.

1.2 **Adipogenic and osteogenic differentiation assays**

DPSCs and PLSCs were seeded at a density of 3 x 10^4 cells/well in a six-well plate and cultured for 48 h in basal medium consisting of α-MEM supplemented with 10% FBS and 1% antibiotic before osteogenesis or adipogenesis induction. Osteoblast differentiation was induced for two weeks as described by Gopinathan et al., (Gopinathan et al. 2013).
Adipogenesis differentiation was induced with the adipogenic medium for 21 days as described here (Mercado-Rubio et al. 2021). After osteogenic or adipogenic differentiation, the cells were fixed in 4% PFA, rinsed twice with 1X PBS and stained with 0.1% Oil Red O for lipid droplets (Sigma-Aldrich, USA) or 2% Alizarin Red S for calcium deposition. Cells cultured in basal medium without differentiation factors were used as controls.

1.3 **Total RNA extraction and quantitative RT-PCR analysis**

Total RNA was isolated from dental stem cells using a Direct-zol RNA kit (Zymo Research) according to the manufacturer's instructions. For cDNA synthesis, reverse transcription reactions were performed with 1 µg RNA by using the SuperScript First-Strand Synthesis System (Invitrogen) following the manufacturer's instructions. qRT-PCR was performed in triplicate using iTaq Universal SYBR Green Supermix (BIO-RAD) in an Eco Real-time PCR System (Illumina) and analyzed using EcoStudy Software (Illumina). Changes in gene expression were calculated relative to *18S rRNA* using the $2^{-\Delta\Delta CT}$ method (Livak and Schmittgen 2001). The primers used for qPCR are listed in **Supplementary Table 1**.

1.4 **Gene body methylation**

Genome-wide DNA methylation data of DPSCs at different passages from female and male patients were downloaded from Gene Expression Omnibus accession number GSE93134. Sequences were mapped to the human genome reference GRCh38 to visualize the methylated

CpGs on the gene body from the transcription start site to the transcription end site by using the software of the Broad Institute, Integrative Genomics Viewer (http://software.broadinstitute.org/software/igv/).

1.5 **Statistical analysis**

Data are presented as the mean ± standard deviation. Experiments were repeated three times. Significance was analyzed using SigmaPlot software. One-way analysis of variance followed by Tukey's test was used to determine the significant differences among the groups.

Supplementary References

Gopinathan G, Kolokythas A, Luan X, Diekwisch TG. (2013). Epigenetic marks define the lineage and differentiation potential of two distinct neural crest-derived intermediate odontogenic progenitor populations. *Stem Cells Dev* **22**: 1763-1778. doi:10.1089/scd.2012.0711

Livak KJ, Schmittgen TD. (2001). Analysis of relative gene expression data using real-time quantitative PCR and the 2(-Delta Delta C(T)) Method. *Methods* **25**: 402-408. doi:10.1006/meth.2001.1262

Mercado-Rubio MD, Perez-Argueta E, Zepeda-Pedreguera A, Aguilar-Ayala FJ, Penaloza-Cuevas R, Ku-Gonzalez A, Rojas-Herrera RA, Rodas-Junco BA, Nic-Can GI. (2021). Similar Features, Different Behaviors: A Comparative In VitroStudy of the Adipogenic Potential of Stem Cells from Human Follicle, Dental Pulp, and Periodontal Ligament. *J Pers Med* **11**. doi:10.3390/jpm11080738
